# Modelling the impact of chest X-ray and alternative triage approaches prior to seeking a tuberculosis diagnosis

**DOI:** 10.1186/s12879-019-3684-1

**Published:** 2019-01-28

**Authors:** Abu A. M. Shazzadur Rahman, Ivor Langley, Rafael Galliez, Afrânio Kritski, Ewan Tomeny, S. Bertel Squire

**Affiliations:** 1 North east medical college hospital, Sylhet, Bangladesh; 2 Centre for Applied Health Research and Delivery, Liverpool School of Tropical Medicine, Pembroke Place, Liverpool, L3 5QA UK; 3 Rede TB, Federal University of Rio de Janeiro, Rio de Janeiro, Brazil

**Keywords:** Triage, Xpert, X-ray, Model

## Abstract

**Background:**

Tuberculosis is a major challenge to health in the developing world. Triage prior to diagnostic testing could potentially reduce the volume of tests and costs associated with using the more accurate, but costly, Xpert MTB/RIF assay. An effective methodology to predict the impact of introducing triage prior to tuberculosis diagnostic testing could be useful in helping to guide policy.

**Methods:**

The development and use of operational modelling to project the impact on case detection and health system costs of alternative triage approaches for tuberculosis, with or without X-ray, based on data from Porto Alegre City, Brazil.

**Results:**

Most of the triage approaches modelled without X-ray were predicted to provide no significant benefit. One approach based on an artificial neural network applied to patient and symptom characteristics was projected to increase case detection (82% vs. 75%) compared to microscopy, and reduce costs compared to Xpert without triage. In addition, use of X-ray before diagnostic testing for HIV-negative patients could maintain diagnostic yield of using Xpert without triage, and reduce costs.

**Conclusion:**

A model for the impact assessment of alternative triage approaches has been tested. The results from using the approach demonstrate its usefulness in informing policy in a typical high burden setting for tuberculosis.

**Electronic supplementary material:**

The online version of this article (10.1186/s12879-019-3684-1) contains supplementary material, which is available to authorized users.

## Background

There were an estimated 1.7 million deaths and 10.4 million new cases of tuberculosis (TB) in 2016 [1]. The standard diagnostic approach for pulmonary-TB relies on sputum smear microscopy (SSM), but published research shows that SSM has limitations [2]. These include accuracy (sensitivity 20–80%) [3], the time taken to complete diagnosis and start treatment (4–20 days) [4] and the related costs [5–7].

New diagnostic algorithms to improve accuracy and early diagnosis of TB, including detection of resistance to TB drugs, are required [8]. Xpert MTB/RIF (Xpert) is a rapid, automated molecular test that can detect TB with higher sensitivity (83 to 92%) and, at the same time, resistance to rifampicin [9]. However, due to the high cost per test, implementation of Xpert in many countries is limited [5]. As an example, Porto Alegre City in Brazil is a high prevalence setting for TB with high levels of HIV-coinfection [10]. Data collected in 2011 as part of the Policy Relevant Outcomes from Validating Evidence on Impact (PROVE-IT) study in Brazil [11] showed the prevalence of TB among presumptive-TB cases at primary health care facilities was 15.8% with HIV coinfection at 44.8%. Recent research showed 4.7% of smear-positive pulmonary-TB cases were multi-drug resistant [12]. Porto Alegre is a city where implementation of Xpert could have a significant impact on reducing the TB burden. Currently all presumptive-TB cases are diagnostically tested for TB. If a nurse or clinician could identify using characteristics of the patient and their symptoms (triage) some of the individuals that do not have TB, then the number of diagnostic tests conducted could be reduced, saving cost and speeding up access to TB treatment for those where it is needed [13, 14].

Operational modelling has been used to project health system and patient impacts of introducing new diagnostic algorithms [15–17]. Such an approach could be used to evaluate the impact of triage prior to seeking a diagnosis. This study investigates the use of operational modelling to predict the impact of seven potential alternative approaches to triage (including no triage – base case), with or without X-ray and in combination with the Xpert diagnostic test. The projected outcomes were compared to a base case of sputum smear microscopy without triage or X-ray prior to diagnostic testing.

## Methods

### Operational model

**Fig. 1:**
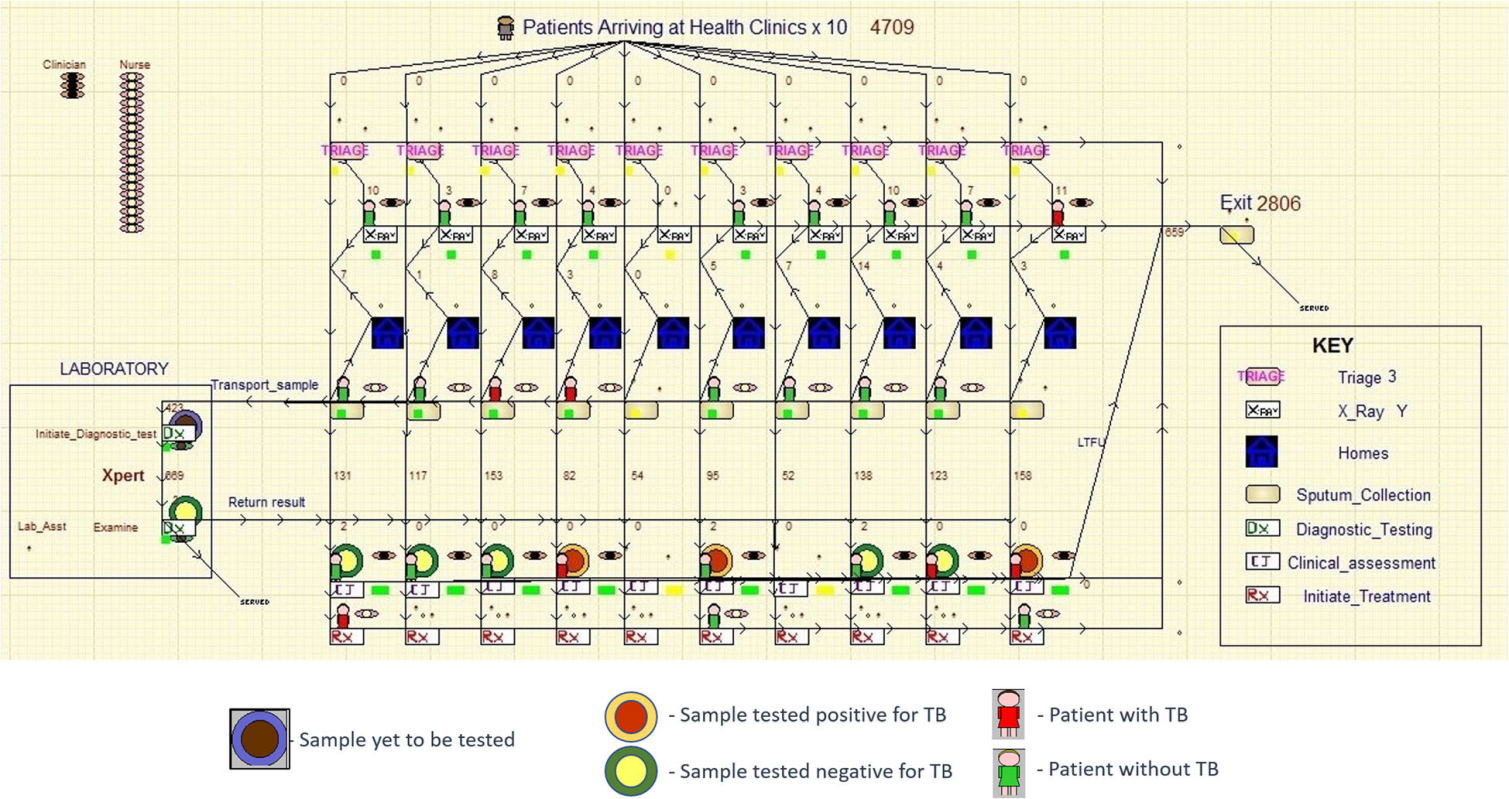
Example screenshot of operational model of TB diagnostics in Porte Alegre. The screen shot of the model illustrates presumptive-TB cases arriving at 1 of 10 health clinics where they undergo the triage test followed in some cases by X-ray. Patients who are triage positive then proceed for sputum collection. When microscopy is used for diagnosis the patients then go home and return the next day with a second sputum sample. Sputum samples are tested in the laboratory using either microscopy or Xpert MTB/RIF. A red patient icon indicates the patient has active TB and a green icon indicates a patient with no TB. Sputum samples and results are shown as circles. Circles with brown centres represent initiation of the diagnostic test and TB positivity unknown, red and yellow centres indicate samples that tested positive and negative respectively. Patients who are tested positive, undertake initiation of TB treatment and those who are tested negative go for clinical assessment and then TB treatment if clinically diagnosed. Some patients are also shown as lost to follow up (LTFU) and no treatment is initiated. Three types of resources are also shown in the model to represent Clinicians (Orange and Black), Nurses (Pink and Yellow) and Lab Assistants (Green and Brown)

For this study an operational model was chosen as it could be designed to fully represent the current and potential future patient pathways for diagnosis in Porto Alegre using a visual and interactive model that could engage decision makers. The activities of triage, sputum collection, diagnosis, clinical assessment and treatment initiation were modelled. Waiting areas for patients along with the human resources required for each activity were represented in the model. A snap shot of the screen layout for the developed model is shown in Fig. 1 including a description of the patient pathways. The model was developed using the discrete event simulation (DES) package – WITNESS® [18]. There are five key elements that need to be defined within any WITNESS® DES model. The first of these are ‘entities’ representing either people or objects moving around a process. These entities have ‘attributes’ which can be used to represent either static or changing features of the entity (e.g. quantity, TB status, patient unique identifier, and time in a particular process). Entities travel through ‘activities’ (representing processes where time and resources are involved) and ‘queues’ (representing waiting areas before activities). Activities can be associated with ‘resources’ such as staff. More detail on the structure of the model is in the online appendix. The dynamic and visual representation of the process facilitated validation and calibration of the model.

Data from January to December 2012 were collated for Porto Alegre City in Brazil, sourced in part from the PROVE-IT study [11] (Table 1) to populate and calibrate the model.

**Table 1:** Input data

Parameter	Value (95% CI)	Source
Mean number of presumptive TB cases per day	44 (34,55)	Data collated from primary health care facilities in Porto Alegre for the PROVE-IT trial [11]
TB prevalence amongst presumptive-TB cases	15.8% (14.6, 17.0%)	
HIV prevalence in TB cases	44.8% (40.4, 49.2%)	
HIV prevalence in no TB cases	27.3% (25.8, 28.8%)	
Sensitivity - Smear microscopy for HIV-positive	45% (38, 52%)	Boehme *et al* [35]
Specificity - Smear microscopy for HIV-positive	100% (99, 100%)	
Sensitivity - Smear microscopy for HIV-negative	72% (69, 75%)	
Specificity - Smear microscopy for HIV-negative	99% (99, 100%)	
Sensitivity - Xpert for HIV-positive	82% (77, 87%)	
Specificity - Xpert for HIV-positive	99% (98, 100%)	
Sensitivity - Xpert for HIV-negative	92% (90, 94%)	
Specificity - Xpert for HIV-negative	99% (98, 99%)	
Sensitivity - Clinical judgement for HIV-positive	49%	Estimated from reported TB case volumes in Porto Alegre and assumed sensitivity/specificity of Smear microscopy
Specificity - Clinical judgement for HIV-positive	90%	
Sensitivity - Clinical judgement for HIV-negative	77%	
Specificity - Clinical judgement for HIV-negative	90%	
Sensitivity of X-ray for abnormalities suggestive of active TB	87% (79, 95%)	WHO [26]
Specificity of X-ray for abnormalities suggestive of active TB	89% (87, 92%)	
Estimated unit cost per test – Microscopy	US$7.20	Estimates provided by TB research staff working with the TB program in Porto Alegre
Estimated unit cost per test – Xpert	US$ 17.80	
Estimated unit cost per test – X-ray	US$ 6.00	
Estimated % of presumptive-TB cases LTFU	10.0%	
Estimated cost to treat TB case in Brazil	US$840	Laurence *et al* [36]
Estimated cost to treat MDR-TB in Brazil	US$6313	

### Triage tests

Seven potential triage approaches for TB diagnosis were identified using literature review and expert interviews. These triage approaches were selected on the basis that they made use of information that would be readily available prior to performing a diagnostic test. For example, personal data such as age, HIV-status, and tobacco usage, and clinical symptoms such as cough, fever, chest pain, weight loss, haemoptysis and other respiratory symptoms [19, 20]. In addition, approaches that could combine this information to generate a predictive algorithm for active TB were considered [21]. Algorithms such as a clinical score [22] developed using regression models or an artificial neural network (ANN) [23, 24] were identified. For these approaches, some computation would be necessary by the diagnosing health professional using a scorecard where points are allocated to individual or combinations of characteristics. Six potential alternative triage approaches with estimated sensitivity and specificity for active pulmonary-TB were identified (Table 2). For comparison purposes these included the theoretical target product profiles (TPP) for a triage test proposed by Denkinger et al. [25].

**Table 2:** Optional Triage approaches and key characteristic assumptions

Triage label	Description of triage approach	Sensitivity	Specificity	Additional cost per test^b^
T1- Base case	No triage			
T2- Cough 1 week	Respiratory symptom of cough > 1 week [18]	88%	19%	US$0
T3 Cough 3 weeks	Respiratory symptom of cough > 3 weeks [18]	61%	51%	US$0
T4- Clinical Score	Scorecard based on aggregating scores assigned to respiratory symptoms including chest pain, cough, sputum expectoration, hemoptysis, night sweats, fever, shortness of breath and weight loss [18].	83%	52%	US$2
T5- ANN	Artificial Neural Network (ANN) based on using a multilayer perceptron (MLP) approach [19] to infer the probability of a patient having active pulmonary-TB from personal data and clinical symptoms i.e. age, gender, cough, fever, weight loss, smoker, night sweats, hospitalisation, chest pain, dyspnea, and hemoptysis.	98%^a^	32%^a^	US$2
T6- TPP (optimal)	A theoretical optimal target product profile (TPP) as proposed by Denkinger et al. [21]	95%	80%	US$2
T7- TPP (minimal)	A theoretical target product profile (TPP) with the minimum characteristics required to be useful as proposed by Denkinger et al. [21]	90%	70%	US$2

^a^the sensitivity and specificity figures are taken from unpublished research in Brazil

^b^The additional cost per triage test is assumed to be low as the characteristics are those which clinicians will already consider today. An additional allowance (US$2) has been made if some computation is required in line with the costs proposed by Denkinger *et* al [20] for the TPP’s

Additional data used in the model is detailed in the online appendix.

**Fig. 2:**
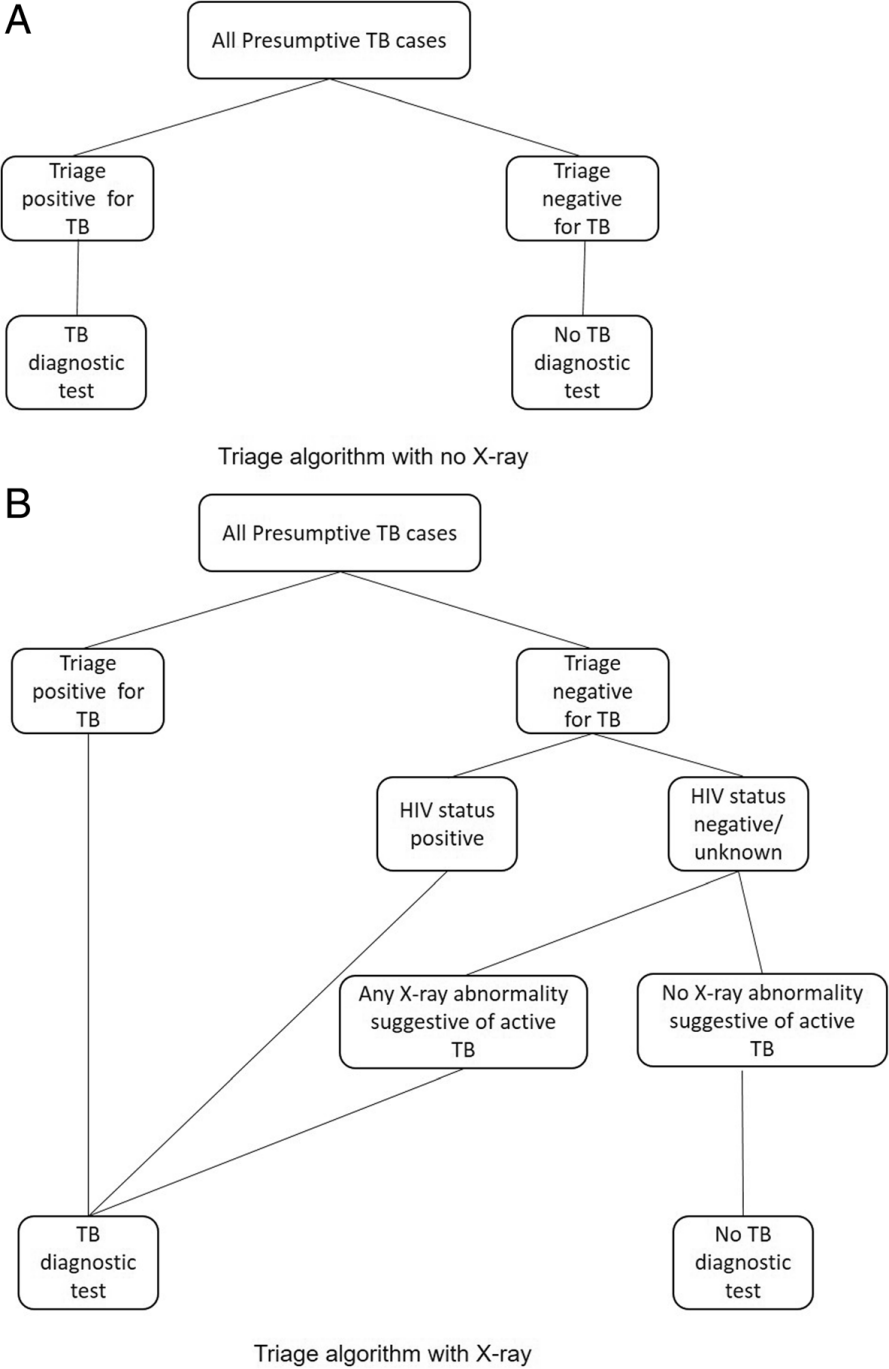
Alternative presumptive-TB algorithms for triage and X-ray prior to TB diagnostic testing

Chest X-ray is also an approach commonly used by programmes for triage. Therefore, we also considered X-ray in combination with other triage algorithms as a tool to ensure all patients with X-rays suggestive of TB would receive a diagnostic test [26, 27]. We modelled an X-ray algorithm as a potential add-on to triage for HIV-negative (or unknown status) presumptive-TB cases with any abnormality suggestive of active TB. In these scenarios, it was assumed all HIV-positive presumptive-TB cases would go for laboratory testing due to the difficulty of detecting TB using X-ray in HIV patients (Fig. 2).

### Diagnostic algorithms

In Porto Alegre two alternative diagnostic algorithms were considered for testing of presumptive TB cases. Presumptive-TB cases were defined as patients who present with symptoms or signs suggestive of TB at primary health care facilities in Porto Alegre City [28]. The first diagnostic algorithm available was sputum smear microscopy based on two samples collected on different days followed by a clinical assessment for smear negative cases. The second diagnostic algorithm was based on a single sputum sample tested using Xpert MTB/RIF followed by clinical assessment for Xpert negative cases. Both these algorithms were modelled.

### Model outputs

The model projected the impact of introducing each alternative triage approach prior to the diagnostic test. Note the context here is triage of patients seeking a TB diagnosis rather than active case finding. The impacts on patients sent for diagnosis, TB cases identified, false positive diagnoses, time to diagnosis and resource usage were projected using the model. The case detection rate (defined as the number of patients with active TB disease that are diagnosed (bacteriologically confirmed or clinically diagnosed) and start treatment, divided by the number of presumptive TB cases with active TB disease). 95% confidence limits were calculated for the key outputs. Sensitivity analysis to the prevalence of TB in presumptive-TB cases was conducted.

### Costing

A unit cost to the health system was estimated for each test including triage, X-ray and diagnostic tests (Tables 1 and 2). The unit costs included staff time, consumables, cartridges, slides, running costs and equipment depreciation. They did not include fixed overhead costs (e.g. space) as these were assumed unchanged between tests. The additional cost per triage test was assumed to be low as the characteristics are those which clinicians will already consider. The X-ray and diagnostic costs were taken from the Prove-IT study in Brazil [11] which used an activity-based approach to take into account cost drivers relating to physical infrastructure, human resources, supplies (chemicals, reagents and consumables), and transport. The ratio of the increase in health system costs divided by the benefits in number of true TB cases starting treatment was also assessed as a measure to compare alternative triage approaches.

## Results

Summary projections for the impact of introducing Xpert in Porto Alegre, with or without triage, for each of the modelled scenarios are shown in Tables 3 (without X-ray) and 4 (with X-ray).

**Table 3:** Model projections with Xpert as the diagnostic tool for each triage option when no X-ray available for triage (Fig. 1a). (Base Case – Microscopy diagnostic tool and no triage)

Diagnostic & triage options	Presumptive TB cases receiving diagnostic test per yr.	True TB cases ^b^ starting treatment per year and case detection % ^c^	False TB cases ^d^ starting TB treatment per year	Time between starting triage and receiving diagnosis (days)	Additional true TB cases starting treatment over base case per year ^a^	Additional cost compared to base case per year ^a^ (US$ 000 s)	Cost per additional true TB patient diagnosed and treated n(US$) ^a^
Microscopy No Triage (base case)	10,281	1238 75%	543	6.0	0	0	0
Xpert No Triage	10,284	1375 83%	419	5.0	137 (57, 217)	581 (555, 607)	4242 (1372, 7111)
Xpert T2 Cough 1wk	8411	1197 72%	340	4.0	−41 (−118, 36)	233 (223, 244)	No benefit over base
Xpert T3 Cough>3wks	5183	878 53%	193	2.4	−360 (− 439, −281)	− 393 (− 410, − 375)	No benefit over base
Xpert T4 Clinical score	5470	1148 70%	191	2.5	−90 (−166, −14)	−51 (−53, −49)	No benefit over base
Xpert T5 ANN	7469	1369 82%	285	3.5	131 (39, 223)	367 (351, 384)	2805 (907, 4703)
Xpert T6 TPP optimal	3290	1320 80%	75	1.5	82 (2, 162)	−49 (−52, −47)	− 604 (−1012, − 195)
Xpert T7 TPP minimal	4046	1245 76%	121	1.8	7 (−74, 88)	−54 (−56, −52)	Minimal benefit over base case

^a^Numbers in brackets represent 95% confidence limits

^b^True TB cases include both bacteriologically confirmed and clinically diagnosed cases that have TB

^c^Case detection rate calculated as the number of true TB cases identified through the complete triage and diagnostic algorithm, divided by the number of TB cases in the presumptive-TB case population calculated from the assumed TB prevalence (Table 2)

^d^False TB cases are individuals diagnosed with TB and placed on TB treatment, but do not have TB (false positives)

### Without X-ray – Table 3

For the base case of microscopy without triage, the projected volume of individuals starting TB treatment was 1238 cases per year (75% case detection rate). This included bacteriologically confirmed and clinically diagnosed cases and involved 10,281 patients being tested. The mean time from the patient arriving at the health facility to completing diagnosis was projected to be 6.0 days.

Implementation of Xpert without triage was projected to have a significant impact over the base case. Case detection rate rising to 83% with a projected increase in the number of people with TB starting treatment of 137 (95% CI. 57, 217) per year and an increase in the annual diagnostic cost of US$581 thousand (95% CI. 555, 607). The mean cost per additional TB case treated was projected to be US$4242 (95% CI. 1371, 7111). The mean time to complete diagnosis was reduced by 1.0 day (5 days compared to 6 days).

Implementation of Xpert alongside a triage test of excluding all cases with a cough of less than one week (T2) gave a projected case detection rate of 72% with a reduction in the annual number of patients with TB starting treatment of − 41 (95% CI. -118, 36) compared to the base case. Therefore, there would be no benefit of this option over the current standard diagnostic approach of microscopy. The same was true for implementation of Xpert alongside a triage test of excluding all cases with a cough less than three weeks (T3) – case detection rate dropping to 53%.

Implementation of Xpert together with a triage test using a clinical score (T4) had a projected reduction in case detection rate to 70% with the annual number of people with TB starting treatment falling by − 90 (95% CI. -166, − 14), so despite the lower cost compared to Xpert without triage, this was not considered a useful intervention.

Using the predicted sensitivity and specificity of the ANN (T5) as the triage test along with Xpert as the diagnostic test showed a significant increase in the projected case detection rate to 82% with an increase in the annual number of TB patients starting treatment of 131 (95% CI. 39, 223). Projected additional health system costs compared to the base case were US$367 thousand (95% CI. 351, 384) compared to US$581 thousand (95% CI. 555, 607) for Xpert without triage. This option therefore both increases case detection compared to the base case and would cost less than roll-out of Xpert without triage.

Using a triage test with the performance of the theoretical optimal TPP (T6) with Xpert was projected to have a positive impact on case detection (80%), cost and time to complete diagnosis. The projected impact on the annual number of people with TB starting treatment was an increase of 82 (95% CI. 2, 162) with a significantly reduced annual health system costs to both microscopy -US$49 thousand (95% CI. -52, − 47) and Xpert without triage.

Implementation of Xpert alongside a triage test with the minimal TPP characteristics (T7) was projected to have no significant impact on case detection rate (76%) or the annual number of TB patients starting treatment compared to microscopy.

**Fig. 3:**
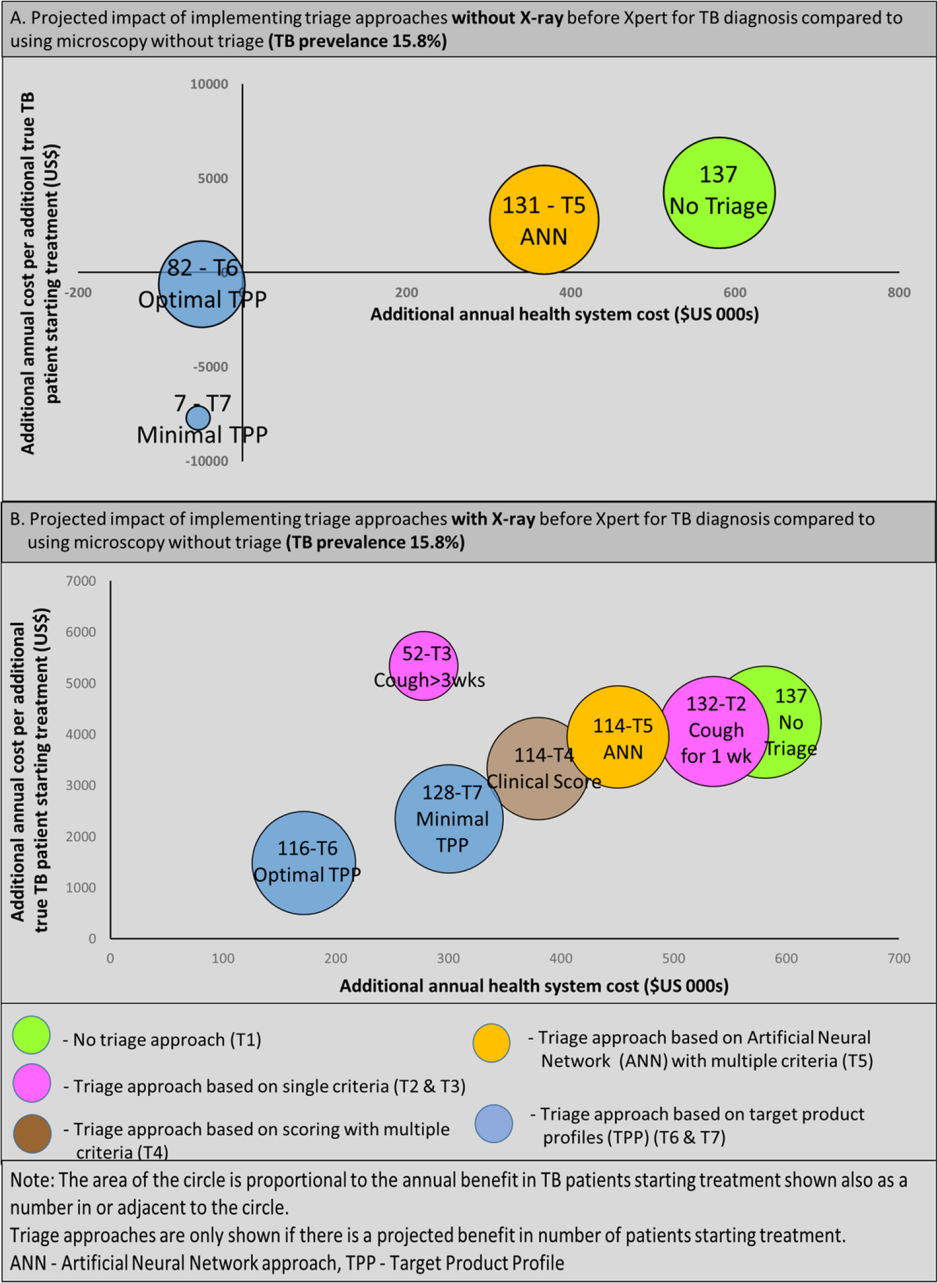
Projections on the impacts of implementing alternative triage approaches (T2-T7) Impacts shown are on health system costs (X-axis), additional cost per additional TB patient starting treatment (Y-axis) and number of additional TB patients starting treatment (size of circle). Graph A is impact of triage without X-ray. Graph B is impact of triage with X-ray

Figure 3a illustrates the projections from the model of each scenario with a positive impact on the number starting treatment compared to the base case. T6 (Optimal TPP) and T5 (ANN) are the most effective options with reduced cost compared to Xpert without triage.

### With X-ray – Table 4

These results are based on the same scenarios as those detailed above, but with X-ray also used as an additional triage tool for HIV-negative (or HIV status unknown) presumptive-TB cases. For HIV-positive patients these options assume all presumptive-TB cases would receive a TB diagnostic test.

**Table 4:** Model projections with Xpert as the diagnostic tool for each triage option with X-ray available for triage (Fig. 1b). (Base Case – Microscopy diagnostic tool and no triage)

Diagnostic & triage options	Presumptive TB cases receiving diagnostic test per yr.	True TB cases ^b^ starting treatment per year and case detection ^c^ %	False TB cases ^d^ starting TB treatment per year	Time between starting triage and receiving diagnosis (days)	Additional true TB cases starting treatment over base case per year ^a^	Additional cost compared to base case per year ^a^ (US$ 000 s)	Cost per additional true TB patient diagnosed and treated (US$) ^a^
Microscopy No Triage (base case)	10,281	1238 75%	543	6.0	0	0	0
Xpert No Triage	10,284	1375 83%	419	5.0	137 (57, 217)	581 (555, 607)	4242 (1372, 7111)
Xpert T2 Cough 1wk	9204	1370 83%	401	4.4	132 (54, 210)	536 (512, 559)	4057 (1313, 6802)
Xpert T3 Cough>3wks	7340	1290 80%	289	3.5	52 (−15, 119)	278 (266, 290)	5345 (1729, 8960)
Xpert T4 Clinical score	7365	1353 82%	296	3.5	114 (38, 190)	380 (363, 397)	3331 (1078, 5584)
Xpert T5 ANN	8477	1352 82%	341	4.0	114 (43, 187)	451 (431, 470)	3952 (1278, 6625)
Xpert T6 TPP optimal	5823	1354 82%	218	2.8	116 (45, 187)	172 (164, 179)	1481 (479, 2483)
Xpert T7 TPP minimal	6367	1366 83%	231	3.1	128 (56, 200)	301 (287, 314)	2349 (760, 3938)

^a^Numbers in brackets represent 95% confidence limits

^b^TB cases include both bacteriologically confirmed and clinically diagnosed cases that have TB

^c^Case detection rate was calculated as the number of true TB cases identified through the complete triage and diagnostic algorithm, divided by the number of TB cases in the presumptive-TB case population calculated from the assumed TB prevalence (Table 2)

^d^False TB cases are individuals diagnosed with TB and placed on TB treatment, but do not have TB (false positives)

Implementation of all the triage approaches with X-ray alongside Xpert as the diagnostic tool would have a significant positive impact on case detection rates compared to the base case (sputum smear microscopy without triage) i.e. 80–83% compared to 75%. As shown in Table 4 and Fig. 3b the projected increase in the annual number of TB patients starting treatment for most of the triage approaches matches the increase projected with no triage when Xpert is the diagnostic tool. The exception is T3 (cough for greater than three weeks) when the increase is smaller. Comparing the results in Table 3 (without X-ray) with the results in Table 4 (with X-ray) shows annual costs increase due to X-ray, an increase in the number of diagnostic tests, and additional treatment costs. However, the projected costs are still below Xpert without triage in all cases. In particular, T4 (clinical score), T5 (ANN), T6 (TPP- optimal) and T7 (TPP-minimal).

### Sensitivity analysis

Variation in the outcomes to the sensitivity and specificity parameters of the triage tests can be seen from the range of different triage tests modelled (i.e. sensitivities ranging from 61 to 98% and specificities from 19 to 80%). Additional sensitivity analyses to input parameters such as TB prevalence and costs of tests were also performed. The results of all the sensitivity analyses are shown in the online appendix. The ranking of options by effectiveness was unchanged by varying these parameters.

## Discussion

Operational modelling can provide valuable predictions of the impact on case detection, health system costs and time to complete diagnosis of alternative triage approaches for TB diagnosis as shown by this study using data from Porto Alegre City, Brazil. The approach can bring together routine and trial data from the current program along with data from published and ongoing research to model current and potential future patient pathways which are critical to understanding patient and health system impacts in relation to cost and time, as well as yield.

The WHO strongly recommends Xpert should be used as the initial diagnostic test in individuals suspected of having TB-HIV coinfection [29]. In Porto Alegre, Brazil, where TB and HIV prevalence are high, the rollout of the Xpert test could have a large effect. However, Xpert is frequently only used as an add-on test to microscopy rather than for initial diagnosis due to its high cost per test. Our study confirms implementation of Xpert would provide a significant benefit over microscopy in terms of the number of patients with TB starting treatment in Porto Alegre City, with case detection rates estimated to increase from 75 to 83%. Introducing a triage approach prior to the Xpert test could reduce costs but would also reduce the number of TB patients starting treatment as some patients that fail the triage test would have TB and would have been identified by the diagnostic test if they had been tested. For example, a triage test based on cough for greater than 3 weeks could reduce the number of diagnostic tests by almost half, but would see many TB cases not being sent for diagnosis leading to case detection falling to 53%. Most of the triage approaches modelled when combined with Xpert did not provide any significant benefit over microscopy as the diagnostic test. However, one triage approach (T5- ANN) was found to significantly increase TB case detection (82% vs. 75%) compared to microscopy and reduce costs compared to Xpert without triage. This approach combines patient and symptom data in a score. This is an encouraging result, but before an ANN approach could be implemented further work is required to develop the appropriate data collection and computation procedures in the diagnostic centre.

The model shows that X-ray combined with a triage approach prior to Xpert diagnostic testing could deliver almost the same case detection rate as would be achieved when no triage is used (i.e. 82–83%). This could be achieved at reduced costs compared to using Xpert for all presumptive-TB cases (i.e. no triage). For example, X-ray combined with the ANN is projected to reduce costs of the roll-out of Xpert to the TB programme by around US$130,000 per year in Porto Alegre city. This would require access to X-ray at diagnostic facilities, which may not be possible in some locations and would require further investigation.

A triage test with the optimal TPP characteristics [25] would also be highly effective but is not available currently. The minimal TPP proposed was not effective as the number of TB patients starting treatment would not be increased.

An additional observation from the modelled diagnostic and triage options is the effect on reducing false positive diagnoses (i.e. the number of individuals placed on TB treatment who do not have TB disease). This is an important observation as the consequences of false diagnosis for TB can be serious for the individual and the TB programme [30]. As expected the use of Xpert as a diagnostic tool compared to microscopy can reduce the rate of false diagnosis particularly when fewer individuals are clinically diagnosed. Our results also indicate the use of triage can lead to reduced false positive diagnosis (Tables 3 and 4), especially if the specificity of the triage test is high (e.g. in triage tests T3, T4, T6 and T7).

Our study was limited by the availability of some data. Assumptions were necessary from the literature and interviews with experts, for example in relation to sensitivity of clinical diagnosis and the new triage approaches as well as associated costs. In addition, it was assumed that the sensitivity and specificity of each of the tests (i.e. triage, X-ray, sputum smear microscopy, Xpert and clinical judgement) were conditionally independent. In particular, this may not be an accurate assumption for triage and clinical judgement as similar criteria may be used by the nurses and clinicians at triage and following a negative diagnostic test. However, this would not be expected to affect the levels of true TB identified through the diagnostic tests. Further analysis of the correlations between tests would be valuable research. We have not tested the approach in low-HIV or high MDR-TB settings, so further research is required here. The modelling methods used in this study could also be used to assess impacts on patient costs [31] and assessing the impact of different strategies for active case finding. Active case finding is likely to be essential if the TB epidemic is to be controlled and is therefore receiving increased focus from the WHO [32] and others [33, 34].

## Conclusions

In conclusion, we have demonstrated that operational modelling as used for this study can provide insights into the impact of alternative triage approaches. In the context of Porto Alegre City, we have shown the introduction of a triage approach alongside Xpert could reduce the TB diagnostic costs of Xpert implementation whilst still significantly increasing the number of patients starting treatment compared to microscopy. Our study indicates that among the optional triage approaches modelled - T5 (ANN) has the greatest potential to improve outcomes whilst controlling costs to the health system. The optimal TPP [25] for TB triage is a theoretical set of performance characteristics for which no triage tools currently exist, but should it become available would be beneficial. Furthermore, adding X-ray as a triage tool for HIV-negative cases (and unknown status) alongside appropriate triage approaches could substantially save costs over Xpert without triage, whilst identifying almost as many cases.

## Additional file

Additional file 1: Online appendix – Jan 2019. Input data. Additional input parameters to the developed model that are not already shown in Tables 1 and 2 in the main manuscript. Namely, Turnaround time distribution observed in the laboratory of Porto Alegre, Triage time assumptions in minutes and Sputum Collection time distribution reported in Porto Alegre. (DOCX 304 kb)

## Abbreviations

AFB: Acid-fast bacilli
ANN: Artificial Neural Network
ART: Adaptive Resonance Theory
CI: Confidence Interval
CJ: Clinical Judgment
DST: Drug susceptibility testing
Dx: Diagnostic tested
FIND: The Foundation for Innovative New Diagnostics
HIV: Human immune deficiency virus
ICER: Incremental Cost Effectiveness Ratio
INH: Isoniazid
LED: Light-emitting diodes
LSTM: Liverpool School of Tropical Medicine
LTFU: Lost to follow up
MDR-TB: Multi drug resistance tuberculosis
MTB: Mycobacterium tuberculosis
NAAT: Nucleic Acid Amplification Test
PROVE-IT: Policy Relevant Outcomes from Validating Evidence on ImpacT
RIF: Rifampicin
Rx: Initiation of Tuberculosis treatment
TB: Tuberculosis
TPP: Target Product Profile
US$: United State dollars
WHO: World Health Organization
XDR-TB: Extensive drug resistance tuberculosis
ZN: Ziehl-Neelsen
