# Supplementary material for: Modelling the impact of chest X-ray and alternative triage approaches prior to seeking a tuberculosis diagnosis
Source: BMC Infect Dis. 2019 Jan 28;19:93. doi: 10.1186/s12879-019-3684-1 (PMC6348624; doi:10.1186/s12879-019-3684-1)
Supplement: Supplementary file 1 — Online appendix – Jan 2019. Input data. Additional input parameters to the developed model that are not already shown in Tables 1 and 2 in the main manuscript. Namely, Turnaround time distribution observed in the laboratory of Porto Alegre, Triage time assumptions in minutes and Sputum Collection time distribution reported in Porto Alegre. (DOCX 304 kb) [file 12879_2019_3684_MOESM1_ESM.docx]

**Online Appendix**

**Modelling the use and impact of triage prior to seeking a tuberculosis diagnosis in the context of Brazil**

Ivor Langley, Rafael Galliez, Abu M Shazzadur Rahman, Afrânio Kritski, Ewan Tomeny, S Bertel Squire.

**Table of Contents**

1. Modelling approach

2. Model structure

3. Sensitivity analyses

4. References

Additional Tables and Figures

**1. Modelling approach**

An operational model to represent the movement of presumptive TB cases and sputum samples through the health system in primary health care facilities in Porto Alegre City was developed. Discrete event simulation (DES) was chosen as the modelling approach. DES is used extensively in the developed world to model processes within health systems^1,2^, and other commercial and public sector situations^3, 4^. However, there is considerably less published material on the use of DES in health studies in low and middle income countries. The chosen modelling software (WITNESS^5^) uses an interactive model building capability which researchers can use without the need for programming skills. The model was designed to be flexible so several different triage and diagnostic algorithms could be represented without major redevelopment. The model was linked to standard databases holding input data and numerical outputs. A visual representation (see Figure 2) of the modelled process was produced by the software which facilitated engagement with, and validation of, the modelled processes. The structure of the model is described below. The input parameters and data sources for the operational component are shown in Table 1 and Appendix Figure 1.

**2. Model structure**

Using the discrete event simulation (DES) approach and the Witness software^5^ a model of the patient pathways in typical TB diagnostic centres in Porto Alegre City, Brazil was developed. In addition, the pathway through the laboratory for sputum samples was modelled. There are five key elements that need to be defined within any Witness DES model. The first of these are ‘entities’ representing either people or objects moving around a process. These entities have ‘attributes’ which can be used to represent either static or changing features of the entity (e.g. quantity, status, patient unique identifier, and time in a particular process). Entities travel through ‘activities’ (representing processes where time and resources are involved) and ‘queues’ (representing waiting areas before activities). Activities can be associated with ‘resources’ such as staff. The key elements used in the model developed for this study are summarised below: -

| Element type | Used to represent |
| --- | --- |
| Entities: | Patients (Presumptive TB cases)  Sputum samples |
| Attributes: | HIV status  TB status  New or retreatment presumptive TB case  Test result  Time triage began  Time diagnostic result available to patient |
| Activities: | Patients   - X-ray - Triage - Sputum collection - Clinical consultation after testing - Clinical Assessment - Initiate treatment   Sputum samples and results   - Transport sputum to laboratory - Initiate diagnostic test (Microscopy or Xpert) - Examine result |
| Queues: | Patients   - Queue for triage/X-ray - Queue for sputum collection - Queue for consultation - Queue for clinical assessment - Queue to initiate treatment - Samples waiting for processing - Results waiting for patient   Sputum sample and results   - Samples waiting for processing - Results waiting for patient |
| Resources | Laboratory technicians  Nurse  Clinician |

The structure of the model followed the patient and sputum sample pathways with the flow between ‘activities’ and ‘queues’ based on rules dependent on the ‘entities’(patients and sputum samples) ‘attributes’.

Patient and sample pathways – Each working day presumptive TB cases visit a diagnostic a health facility. They are categorised as either new cases or retreatment cases depending on whether they have been treated for TB previously. In the model, patients were also categorised as having active TB or not and either HIV-positive or HIV-negative with some knowing their status and some not. If triage is to be used patients will be modelled undergoing a triage test with variable sensitivity/specificity depending on the triage approach. All patients that get a positive result through triage will go onto sputum collection. If X-ray is available all patients with a known positive HIV status will go onto sputum collection even if the triage test is negative. If the patient is HIV-negative or has unknown status they will go onto X-ray with a sensitivity/specificity as per the input parameters (see table 2). If the X-ray is abnormal and suggestive of TB, the patient will be modelled going for sputum collection. All other patients exit the model at this stage as they are diagnosed as very low risk for active TB. If sputum smear microscopy is to be used two samples are modelled being collected on consecutive days. If Xpert is to be used just a single sputum sample would be required. After sputum collection, the sample is modelled being taken to the laboratory for examination whilst the patient waits for the result to be available.

Sputum samples follow a pathway through the district laboratory depending on the patient characteristics, diagnostic technique, and the diagnostic algorithm being used. Two types of diagnostic test were modelled – microscopy and Xpert each requiring different amounts of laboratory technician time. Samples are prepared for the appropriate test; the test is then conducted and a test result assigned based on the sensitivity and specificity of the approach. The time it takes for a single sample to be processed is determined by sampling from an input distribution – see Appendix Figure 1.

The patient is modelled being matched to their result by the clinician, although a percentage will become lost to follow up (LTFU) as they are modelled not returning for their result. If active TB is diagnosed the clinician will start the patient on treatment (Bacteriologically Confirmed TB case). If the patient is negative an additional clinical assessment will be made with some being diagnosed with TB (Clinically Diagnosed TB case) and going onto treatment, whilst others will be diagnosed as not having TB.

For each triage and diagnostic tool scenario the model was run to simulate one year (52 weeks).

**3.Sensitivity Analysis**

Sensitivity analysis of the key input parameters to the outcomes has been completed. Firstly, sensitivity to the accuracy (sensitivity and specificity) of the triage tool. This is shown by the range of potential triage tools investigated with sensitivities ranging from 61% (T3 – Cough greater than 3 weeks) to 98% (T5 – Artificial Neural Network (ANN)) and specificity from 19% (T2 – Cough greater than 1 week) to 80% (T6 – optimal Target product profile (TPP)). The critical value in relation to yield is the Sensitivity as can be seen in the results in Table 3 that show the ranking in yield is in line with the ranking in sensitivity of the triage approach.

Secondly sensitivity to a lower prevalence of active TB in the presumptive TB case population has been conducted. The results of this are shown in Appendix Figure 2 where the predictive outcomes for a presumptive case population with 8% TB prevalence is compared to our analysis with a 15.8% prevalence of TB in the presumptive case population. The ranking for the key output parameters was similar between the two prevalence levels. The one exception was the yield in the T5 – ANN which was adversely impacted compared to T6 – optimal TPP. This appears to be due to the low specificity of T5-ANN (32%) compared to T6 – Optimal TPP (80%) which means when T5 is used as a diagnostic tool many more cases require diagnostic testing leading to operational bottlenecks – particularly if prevalence of TB is low. It must be remembered the optimal TPP (T5) is entirely theoretical and it seems unlikely that a cheap triage test with sensitivity of 90% and specificity of 80% is likely to be available shortly.

Thirdly the sensitivity of the cost of triage for the most promising triage approach – T5 – ANN was conducted. These results are presented in Appendix Figure 3. These results show that if the cost of this triage approach rises to over US$22 then there would be no benefit in reduced costs of the triage/diagnostic algorithm when compared to Xpert without triage.

**4. References**

1. Karnon J, Stahl J, Brennan A, Caro JJ, Mar J, Möller J; ISPOR-SMDM Modeling Good Research Practices Task Force. Modeling using discrete event simulation: a report of the ISPOR-SMDM Modeling Good Research Practices Task Force--4. Value Health. 2012 Sep-Oct;15(6):821-7. doi: 10.1016/j.jval.2012.04.013.

2. Günal M, Pidd M. Discrete event simulation for performance modelling in health care: a review of the literature. J Simulation 2010; 4: 42–51.

3. Mayer G, Spieckermann S. Life-cycle of simulation models: requirements and case studies in the automotive industry. J Simulation 2010; 4: 255–259.

4. Siebers P, Aickelin U, Celia H, Clegg C. Towards the development of a simulator for investigating the impact of people management practices on retail performance. J Simulation 2010; doi: 10.1057/jos.2010.20.

5. WITNESS modelling tool (WITNESS PwE 2.0 Service and Process Performance Edition, Lanner, Redditch,UK, <http://www.lanner.com/en/witness.cfm>

Appendix Figure 1 – Additional input parameter

Turnaround time distribution observed in the laboratory of Porto Alegre


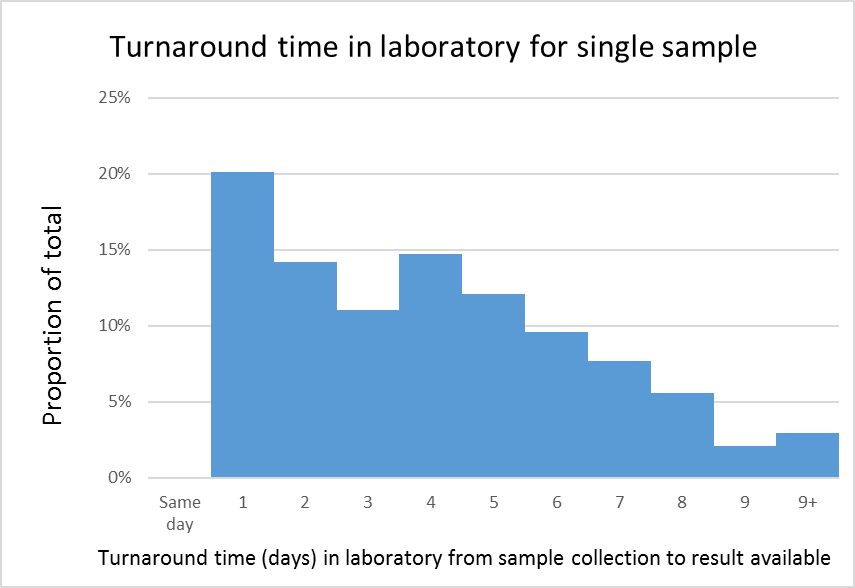


Triage time assumptions in minutes

|  | T1 | T2 | T3 | T4 | T5 | T6 | T7 |
| --- | --- | --- | --- | --- | --- | --- | --- |
| Triage test | No triage | Cough for 1 week | Cough >3 weeks | Clinical Score | Artificial Neural Network | TPP (optimal) | TPP (minimal) |
| Minimum | 0 | 5 | 5 | 25 | 5 | 3 | 5 |
| Maximum | 0 | 10 | 10 | 50 | 10 | 5 | 30 |

Sputum Collection time distribution reported in Porto Alegre

Appendix Figure 2 – Sensitivity of outcomes to the TB prevalence in the presumptive TB case population.


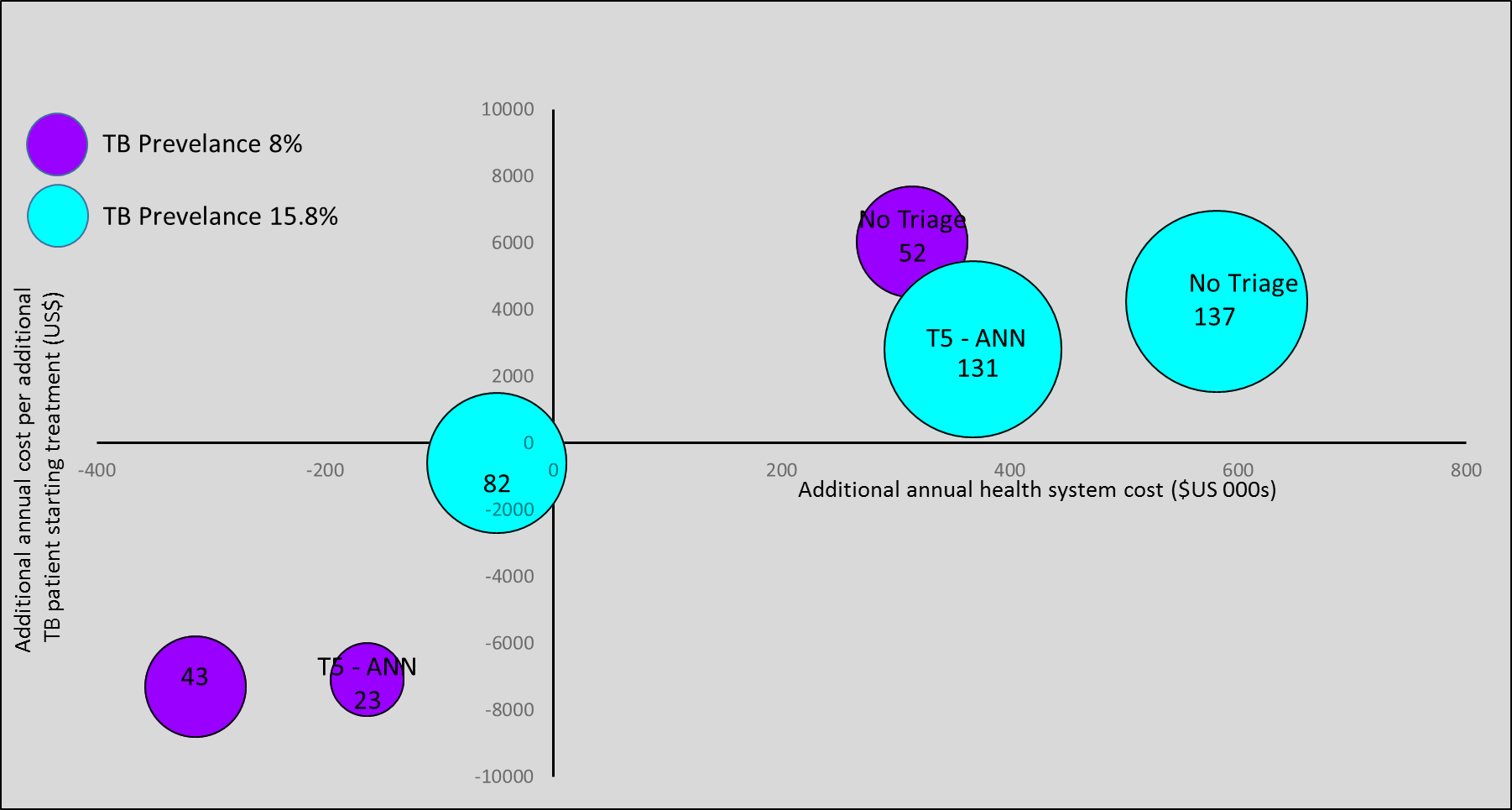


Appendix Figure 3 – Sensitivity of outcomes to cost of triage per presumptive case for T5 – ANN


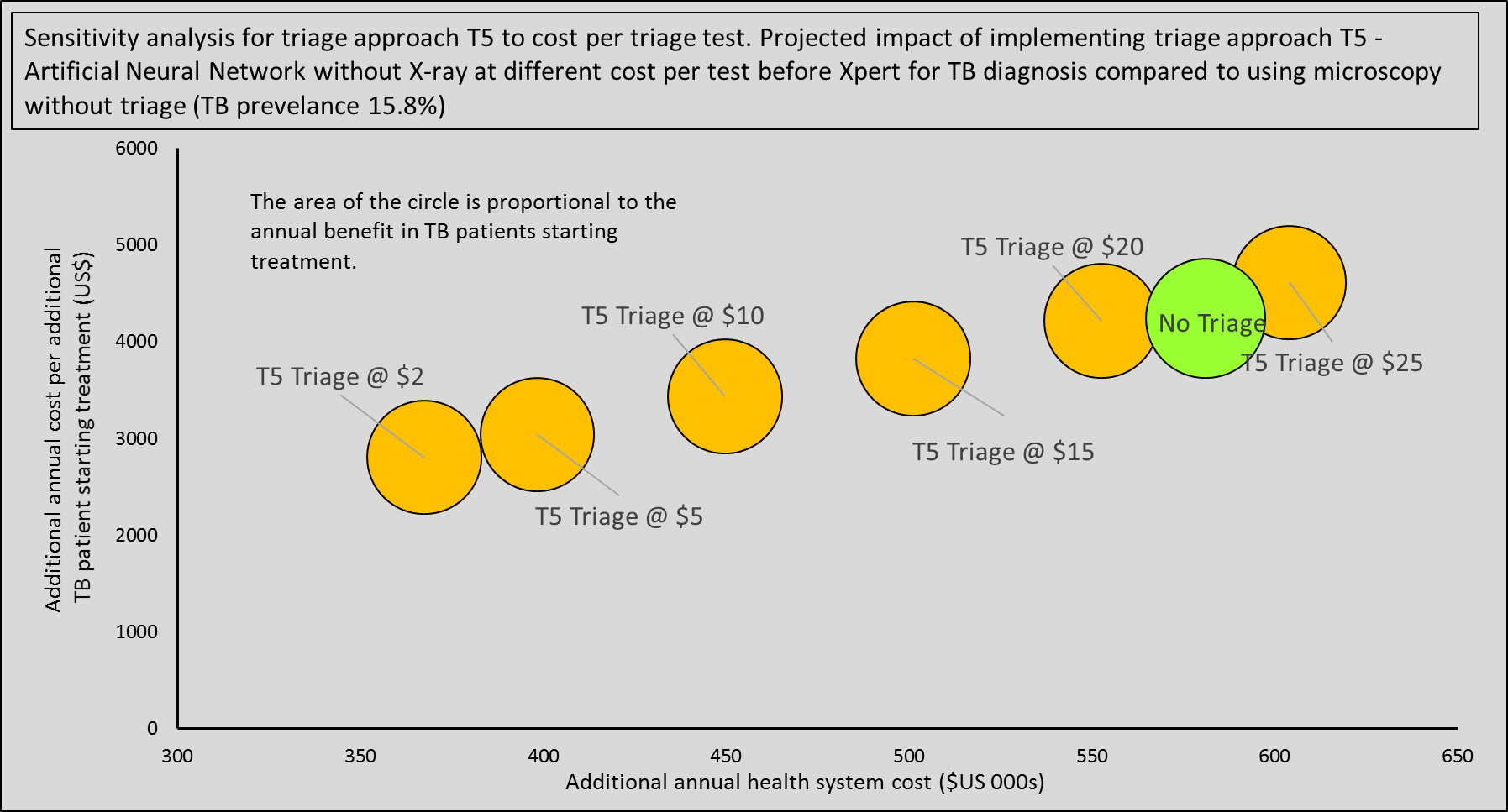


The assumed cost per triage test is shown in US$ in each circle.
